# Are Pyogenic Liver Abscesses Still a Surgical Concern? A Western Experience

**DOI:** 10.1155/2012/316013

**Published:** 2012-02-19

**Authors:** Barbara Alkofer, Corentin Dufay, Jean Jacques Parienti, Vincent Lepennec, Sylvie Dargere, Laurence Chiche

**Affiliations:** ^1^Hepatobiliary Surgery Department, CHU de Caen, 14000 Caen, France; ^2^Medical Statistics, CHU de Caen, 14000 Caen, France; ^3^Radiology Department, CHU de Caen, 14000 Caen, France; ^4^Infectious Diseases, CHU de Caen, 14000 Caen, France

## Abstract

*Backgrounds*. Pyogenic liver abscess is a rare disease whose management has shifted toward greater use of percutaneous drainage. Surgery still plays a role in treatment, but its indications are not clear. *Method*. We conducted a retrospective study of pyogenic abscess cases admitted to our university hospital between 1999 and 2010 and assessed the factors potentially associated with surgical treatment versus medical treatment alone. *Results*. In total, 103 liver abscess patients were treated at our center. The mortality was 9%. The main symptoms were fever and abdominal pain. All of the patients had CRP > 6 g/dL. Sixty-nine patients had a unique abscess. Seventeen patients were treated with antibiotics alone and 57 with percutaneous drainage and antibiotics. Twenty-seven patients who were treated with percutaneous techniques required surgery, and 29 patients initially received it. Eventually, 43 patients underwent abscess surgery. The factors associated with failed medical treatment were gas-forming abscess (*P* = 0.006) and septic shock at the initial presentation (*P* = 0.008). *Conclusion*. Medical and percutaneous treatment constitute the standard management of liver abscess cases. Surgery remains necessary after failure of the initial treatment but should also be considered as an early intervention for cases presenting with gas-forming abscesses and septic shock and when treatment of the underlying cause is immediately required.

## 1. Introduction

Pyogenic liver abscess (PLA) remains a rare disease with a high risk of mortality, up to 19% [1–13]. In recent years, diagnosing PLA and the underlying cause of the abscess has been made easier by modern imaging modalities. PLA management has also changed, with percutaneous drainage and intravenous antibiotics being now considered safe and effective [7, 8, 12]. Even though a nonoperative interventional radiology approach has become the first therapeutic choice for PLA, surgical treatment is still necessary in some cases, although its proper indications remain unclear. Indeed, surgical drainage is associated with increased morbidity and mortality and is often only used as a salvage procedure in cases failed percutaneous treatment [10, 12]. The aim of our study was to retrospectively analyze our monocentric French series of PLA cases and to reappraise the place of surgery in modern PLA management using a recent occidental series.

## 2. Patients and Methods

All of the adult patients treated for a PLA from 1990 to 2009 in the Departments of Infectious Disease, Gastroenterology, and Hepatobiliary Surgery at the University Hospital of Caen were retrospectively reviewed. We excluded all abscesses resulting from a fungal or amebic abscess, infected tumor, or infected liver cyst from the study.

The clinical presentation, past medical history, and microbiological and radiological parameters were obtained from each patient's record. This data included age, gender, presenting symptoms, comorbidities, ASA score, underlying etiology, presence of diabetes mellitus, BMI, presence of malignancy, imaging studies focused on the size and number of abscesses, microbiological findings, and the abscess components (fluid, gas, walls). The size of the abscess was defined as the largest diameter reported on CT. The microbes responsible for the PLA were also noted. The biological parameters examined included hemoglobin and albumin levels, white blood cell count, creatinine level, CRP, fibrinogen, total bilirubin level, and liver enzymes. The etiology of the abscesses was classified into four groups: group 1, biliary; group 2, portal; group 3, cryptogenic; group 4, other (iatrogenic, arterial).

The choice of initial treatment was based on the preference of the attending physician. The treatments were classified as antibiotics alone for 6 weeks (group A), antibiotics and percutaneous treatment (percutaneous needle aspiration (PNA) or drainage) (group AP), or surgical treatment (after percutaneous drainage or not) (group AS). Failure of antibiotic or percutaneous treatment was defined as persistent infection after 3 to 5 days of the chosen therapy. Treating the cause of the abscess was also recorded. Cure was defined as resolution of the infectious syndrome and symptoms at 2 months after initiating treatment.

The factors associated with failure of medical treatment and mortality were studied to evaluate the indications for early aggressive surgical treatment of patients presenting with such factors. Septic shock was defined as a state of combined hypotension, tachycardia, polypnea, oliguria, and altered mental status with a temperature over 38°C or under 36°C. Mortality was defined as death within 30 days or during the concurrent hospital admission.

The data were compiled and analyzed using the SAS v.9.1 software. The continuous numeric data are displayed in tables as means and the categorical data as percentages. The categorical data were compared between groups using either the *χ* ^2^ or Fisher's exact tests. Student's *t*-test was used to compare continuous data. *P* < 0.05 was accepted as significant. Univariate analyses were performed. ROC curves were used to describe the predictive value of certain biological parameters for mortality.

## 3. Results

From January 1990 to December 2008, a total of 103 consecutive patients were treated in the Departments of Hepatobiliary Surgery, Gastroenterology, and Infectious Disease at the University Hospital of Caen, France. Fifty-seven were male, and they had a mean age of 64 years. Fifty patients were ASA II, and the most common comorbidities were active smoking (22.3%) and diabetes mellitus (17.5%). The most common presenting symptoms were hyperthermia (93.0%) and abdominal pain (73.0%). Fourteen patients presented with septic shock, and two patients had an intraperitoneal abscess rupture. The demographic clinical and biological data are reported in Table 1.

All of the biological parameters were representative of a chronic inflammatory state. CRP was elevated in all cases, and its level was over 100 mg/L in 90 patients. We found hypoalbuminemia (<37 g/L) in 97 patients. The microbe responsible could be identified in 71.8% of the cases and was most frequently *Escherichia coli*. This bacterium was isolated in 76.3% of the abscess cultures but in only 41.6% of blood cultures. Anaerobic isolates were found in 24% of the patients.  Table 2 shows an overview of pathogens found in PLA patients and correlates the pathogen found with the abscess etiology.

The imaging characteristics are reported in Table 3. Most of the abscesses were unique (68.3%), measured more than 5 cm (70.7%) and were located in the right liver (58.2%). Nine percent of patients had gas-forming abscesses. Twenty-nine patients (28.1%) presented with a right pleural effusion on admission. The most common cause of liver abscess was biliary tract disorder (45.6%), followed by portal origins (25.2%). Despite thorough investigations, no cause could be found in 18.4% of the cases. The detailed origins of the abscesses are reported in Table 4.

The treatment consisted of antibiotics alone in 16.5% of the cases (group A), as compared to 55.4% with antibiotics and percutaneous treatment (group AP). Overall 41.7% of the patients underwent surgery for PLA treatment (group AS); 28.1% of these cases represented the initial treatment, and 26% were in response to failure of nonsurgical therapy (Table 5).

In group A, 17 patients who were successfully treated with antibiotics alone presented with multiple small abscesses (median size: 3.7 cm). The mean hospital stay was 19. 8 days with a mean duration of treatment of 44, 64 days. Of these patients, 70% were considered cured at 2 months after the initial treatment. Two patients died, one of sepsis and one of the évolution of an underlying cancer.

In group AP, 57 patients were treated with antibiotics and interventional radiology. These treatments failed in 14 patients, who needed additional surgical drainage. The successful initial percutaneous treatment cases involved 26 patients who underwent percutaneous drainage, 7 who underwent percutaneous needle aspiration only, and 6 who required drainage after having had a PNA. One patient needed two PNAs. The PLAs were unique in 72.5% of the cases, with a mean size of 8.7 cm. Walls could be found on imaging in 55% of the patients. The pathogen was identified in 77.5% of the patients, and antibiotic treatment was administered for a total of 50 days. The percutaneous drain was left in place for a mean of 11 days. The mean hospital stay was 25.8 days, and 65% of the patients had recovered at 2 months. Five patients subsequently died.

In group AS, 43 patients underwent surgical treatment. Seventeen (39.5%) of the patients required surgery after antibiotic therapy. Twenty-nine percent of these patients were diabetic. The PLAs were mostly unique (68.3%); the mean size was 4.5 cm, which is slightly larger than the PLAs successfully treated by antibiotics alone. Seventeen percent of these patients presented with septic shock on admission. Most of the abscesses originated from biliary tract disorders (76%). All of the patients were cured, except for one patient who died.

Fourteen (32.5%) group AS patients required surgery after failure of percutaneous treatment. Their abscesses were unique in 57% of the cases and measured more than 8 cm. A total of 35% of the abscesses were of biliary origin, and 35% were of portal origin. Surgery resolved all symptoms and helped find the cause of the abscess in one-third of the patients.

Twelve group AS patients (28%) underwent surgery as the initial treatment. One-third were diabetic. Eighty-three percent of the abscesses were unique, with a mean size of 9,14 centimeters. Two presented with intraperitoneal rupture. Twenty-five percent presented with septic shock. Sixty-seven percent of the PLAs resulted from biliary tract disorders. One patient died of pulmonary embolism 5 days after the surgery. All of the other patients had recovered completely by 2 months. Mean hospital stay was 37 days.

Of the patients needing surgery, 79% underwent simple drainage of the abscess cavity with large abdominal drains. Hepatectomy was necessary in 21% of the patients; 2 underwent major liver resections, and the majority only required minor liver resection, mainly segmentectomies.

The causes of the abscesses were treated in 80% of the cases. In total, 64.6% of the patients required a surgical procedure to treat the cause, which was performed after resolving the PLA in 27.5% of the patients. Forty-two patients underwent surgical treatment of the PLA cause during the same hospital stay; this treatment consisted of 21 cholecystectomies, 8 colectomies, 4 appendectomies, 3 choledocojejunostomy resections, 1 gastrectomy, and 1 nephrectomy.

In the univariate analyses, the only significant factor associated with the surgical PLA treatment was BMI (26.8 versus 24.2; *P* = 0.03). Diabetes mellitus and septic shock were more likely to be associated with surgery, but neither reached significance (*P* = 0.08 for both). The factors associated with failed medical treatment and requiring surgical therapy were gas-forming PLA (*P* = 0.006) and severe sepsis or septic shock (Table 6).

The overall mortality was 8.7%. Four men and 5 women, with a mean age of 72 years, died during the study period. Five patients died of causes directly related to the PLA, and 4 died of thromboembolic complications or of cancer evolution. Severe sepsis and septic shock were the most common causes of death.

In the univariate analyses, the factors associated with mortality were a high ASA score (*P* = 0.02), neoplastic PLA origin (*P* = 0.047), and right pleural effusion (*P* = 0.018). According to the *χ* ^2^ test, however, the PLA cause was not significantly associated with mortality. The ROC curves constructed for the albumin level and the hemoglobin level (chosen as a sign of severity of the sepsis) showed that an albumin below 21 g/L predicted mortality with a sensitivity of 60% and a specificity of 80%. A hemoglobin level below 9,5 g/dL predicted mortality with a low sensitivity (40%) but a high specificity (90%).

## 4. Discussion

This is the first monocentric French PLA series concerning since 1988. The series reported in the literature after 1988 have mainly been from Asian countries, with the largest series (483 patients) being reported by Chou et al. in 1997 [1]. 

Our series of 103 PLA patients found epidemiology that was similar to that of the other series (Table 7), with a slight male predominance. A PLA diagnosis was associated with certain clinical and imaging features, with fever and abdominal pain being the most common (Table 7). In our series, the microbiological isolates found in cultures were different from those reported in Asian studies; the most common isolates were *E. coli* and *Streptococcus* spp., whereas *Klebsiella pneumoniae* is often the second-most common PLA pathogen isolated in Asian series [1, 8, 10–12, 14–16]. Indeed, a recent Spanish series of 84 patients reported that *Streptococcus* spp. was by far the most commonly found (40.5%) microbe [17].

As for diagnosing PLA, two biological criteria emerge from this series. The principal (100%) abnormal biochemical finding was an elevated CRP. This finding is interesting because it is the only highly sensitive indicator and because it was over 100 mg/L in 87% of the cases. Leukocytosis, although reported elsewhere as being the most common PLA finding, was only present in 74.7% of the cases in our series [1, 5, 8, 10]. 

This series confirms that biliary tract diseases are the major cause of PLA [3, 8, 12, 16, 18, 19]. Portal phlebitis from a bowel infection was the second-most frequent cause, at 25.2%. This etiology was the most commonly found in a previous study [20]. Interestingly, our series found fewer cryptogenic PLAs than previous series. Indeed, only 18.4% of the PLAs were cryptogenic, as compared to more than 40% in earlier studies [8, 10, 15]. This lower percentage may have been due to the more extensive etiological research that used more precise and sensitive imaging, as has been suggested by Cohen et al. [21], who found that only 10% of abscesses to be cryptogenic. A higher rate of identified pathogens is also helpful in determining the PLA etiology [22].

Most of our patients (55%) were treated using a combination of antibiotics and percutaneous treatment (aspiration or drainage). This combination is currently the PLA treatment of choice because it is a safe and effective option that offers a low-risk approach to these often critically ill patients [4, 10, 11, 19, 23–27]. However, percutaneous therapy frequently fails, with rates ranging from 15% to 36% (similar to the 26% rate found in our series) [1, 11, 23, 28]. 

The causes of failed PLA treatment include multiple abscesses with multiple loculations [1, 12], large abscesses (>5 cm) containing thick pus with necrotic tissue, issues directly linked to the drainage catheter (blockage, slippage, and chest empyema due to transpleural drainage), and the technical difficulties associated with performing safe drainage. In our experience, there were significantly more gas-forming abscesses (*P* = 0.006) and patients in septic shock (*P* = 0.02) among the cases of percutaneous treatment failures. Our study represents the first occasion that these two criteria have been linked to failed medical treatment. Chou et al. [6] specifically studied gas-forming abscesses and concluded that they are more severe, often presenting with septic shock and are associated with higher mortality than non–gas-forming abscesses (27.7%). Their treatment algorithm starts with mandatory percutaneous treatment but does not recommend surgery as an initial treatment. 

As in other series, we found that PLAs linked to biliary tract disorders failed to respond to medical therapy more often than other PLAs. Indeed, effective biliary drainage is essential to treating such PLAs, and PLA drainage alone is not enough to achieve a cure [29–31].

As a result of the large variety of medical and interventional treatments, surgery is usually only considered in cases of failed percutaneous treatment; therefore, the two approaches can be considered complementary and not competitive [4–6]. Surgery has indications for initial PLA treatment and should not be considered only a secondary therapeutic option in cases of failed percutaneous treatment. It is widely accepted that surgery is mandatory in cases where these is abscess rupture into the peritoneal cavity. Surgery is also necessary if drainage cannot be performed. In addition, multiloculated abscesses are often better treated by surgery that completely breaks down of all the loculations [12, 32].

Surgery is also discussed in other circumstances, depending on the mortality-associated factors involved. Some authors agree that surgery should be the first-line therapy in cases of high mortality risk to improve the chances of survival in these critically ill patients [12, 28]. 

For these reasons, it is important to study the factors linked to mortality. Mortality has improved in recent years thanks to more rapid diagnosis, more effective antibiotics, and the development of percutaneous procedures. Our series had an overall mortality rate of 8,7%, which is on the low end of the rates reported in the literature (0–46%) (Table 7). The factors linked to mortality in our series' univariate analyses were a high ASA score (*P* = 0.02), a malignant PLA origin and the presence of a pleural effusion (*P* = 0.018). The pleural effusion is an interesting factor, as has been previously reported [33], and is probably secondary to the importance of right upper quadrant sepsis. The other factors that have been linked to mortality are severe sepsis and shock, high leukocytosis, low hemoglobin, increased creatinine, elevated blood urea nitrogen and hypoalbuminemia [11, 17]. Gas-forming abscesses have also been independently associated with mortality [6, 14, 34].

The general condition of the patient is an important factor that affects mortality, although it is difficult to evaluate. Patient health indices have been developed in consultation with anesthesiologists, and statistical analyses (univariate and multivariate) have reached different conclusions. Some teams have reported that an APACHE score >15 is significantly associated with mortality [5, 14, 16], and some authors agree that these patients are better served by surgical exploration [1, 8]. Similarly, Lok et al. found in a recent series that the existence of >2 comorbid illnesses was associated with mortality in a univariate analysis [15].

Therefore, two contrasting perspectives on PLA can be noted. One has been expressed by Mishinger et al., who maintain that patients with high mortality risks based on their APACHE score should not undergo surgery as a first-line treatment but only percutaneous drainage to attempt to resolve the PLA by the least invasive methods possible [5]. Their indications for surgery consist of failed medical treatment or PLA ruptures into the peritoneal cavity. By contrast, our team and others [16, 35] have proposed a more aggressive therapy, especially if the patient is critically ill or has high mortality risk. This position is based on the percentage of failed percutaneous drainage attempts, which was 26% in our series and 27,8% for Tan et al. [12]. Such a high failure rate for critically ill patients implies delayed effective therapy, which increases the mortality rate [10, 35]. We think that an aggressive approach, such as an initial surgical procedure, should be the treatment of choice in patients with poor prognosis or failure of percutaneous drainage, especially given that the mortality rate after surgery, which was high in the 1980s, has decreased as hepatic surgery has improved. Indeed, in our series, we observed a 0% surgical mortality rate.

The experience accumulated through these different series helps to distinguish two types of situations leading to surgical PLA management. The first set of indications is linked to the risk of failed percutaneous treatment and include gas-forming abscesses, multiloculated abscesses, and septic shock at presentation. The second set of indications is linked to factors associated with high mortality: an elevated ASA score, an APACHE score > 15, severe sepsis (leukocytosis, albumin < 21 g/dL, hemoglobin < 9,5 g/L), pleural effusion, and a malignant PLA origin.

In conclusion, even though percutaneous treatment combined with antibiotics is a safe and effective PLA treatment, PLA remains a surgical concern. We suggest that critically ill patients with severe sepsis associated with gas-forming or multiloculated abscesses on imaging should undergo surgery as a first-line treatment. Surgery is particularly appropriate for patients with low albumin and hemoglobin and a high ASA score. Patients with a right pleural effusion should also be considered at high risk for mortality and therefore likely to benefit from aggressive therapy.

## Figures and Tables

**Table 1:** The demographic, clinical, and biological data.

Age	64,4	15–94
Sex ratio (M : F)	1,24	1,24

	Number of patients	Percentage

BMI > 30	16/103	15.50%
ASA > 2	68/103	66%
Immunosuppression	2/103	1.90%
Chronic alcohol intake	16/103	15.50%
Clinical presentation		
Fever > 38,5°C	95/102	93%
Abdominal pain	74/101	73%
Chills	47/86	54%
Vomiting	31/102	30%
Jaundice	13/102	12.60%
Hepatomegaly	8/102	7.70%
Biology		
CRP > 6 mg/L	103/103	100%
Fibrinogen > 4,5 g/L	87/91	95.60%
Albumin < 37 g/L	97/103	94.30%
Proteins < 60 g/L	32/95	33.60%
Leukocytes <4000/>10000	74/96	74.70%
Hemoglobin < 12 g/dL	66/99	66.60%
Platelets <150,000/>500,000	27/96	28.10%
GGT > 38 UI	89/95	93.60%
ALAT > 34 UI	87/95	60.40%
Bilic > 3 *μ*mol/L	86/103	83.5%

**Table 2:** The pathogens identified in the liver abscesses.

	Percentage of patients
Pathogen identified	71.80%
*E. coli*	39.20%
*Klebsiella*	9.50%
*Streptococcus*	36.50%
*Fusobacterium*	6.70%
*Staphylococcus *	5.40%
Multiple	35.10%

**Table 3:** The imaging characteristics.

		Number	Percentage (%)
Number	Unique	69/102	68.30%
	Multiple	27/102	26.70%
	Diffuse form	6/102	6%

Size (max)	<5 cm	30/103	30.9
	5–10 cm	46/103	44.6%
	>10 cm	27/103	24.5%

Localization	Right lobe	60/103	58.20
	Left lobe	25/103	24.20%
	Bilobar	18/103	17.40%

Walls		36/103	36%

Gas-forming abscess		9/100	9

**Table 4:** The origin of liver abscesses.

Etiology	Percentage (%)	Benign lesions	Underlying cancer
Biliary	45.60%	12.6% cholecystitis 12.6% dysfunctional hepaticojejunostomy	4.8%
Portal	25.20%	14.5% colic diverticulitis	2.8%
Arterial	5.80%	dental abscesses	
Cryptogenic	18.40%		

**Table 5:** The pyogenic liver abscesses treatments used.

	No. of patients
Group A: antibiotics	17
Group AP: percutaneous treatment	57
Group AS: surgery	43

**Table 6:** Surgical versus medical treatment.

	No surgery	Surgery	*P*
BMI	24.2	25.8	0.25
ASA	2.01	2.24	0.16
Diabetes	9.40%	20.69%	0.15
Jaundice	13.20%	1.70%	0.74
Anemia	67.90%	62.90%	0.66
Hyperleukocytosis	79.50%	71.40%	0.42
Hypoalbuminemia	97.70%	96.30%	0.72
Number	1.33	1.31	0.78
Size	7.04	7.54	0.56
Walls	48.08%	32.14%	0.16
*Gas-forming PLA*	**1.80%**	**21.40%**	**0.006**
Pleural effusion	22.60%	31.03%	0.41
*Septic shock*	**3.70%**	**24.10%**	**0.008**

**Table 7:** A PLA literature review.

Series	Country	Year	Age	Male patients %	Number of patients	Fever %	Abdominal pain %	Unique PLA %	Right lobe %	Gas %	Mortality %
Farges (1)	France	1998	51	47	46	75	55	?	?		24
Branum (2)	USA	1990	53	52	73	78	80	59	70		19
Stain (3)	USA	1991	47	59	54	45	14	57	59		2
Mischinger (4)	Austria	1994	51	65	46	80	54	68	74		17
Chou (5)	Taiwan	1995	56	58	424	92	55	70	65	20	16
Huang (6)	USA	1996	55	57	153	67	89	52	63		31
Chu (7)	Hong Kong	1996	60	57	83	70	53	?	69		18
Chou (13)	Taiwan	1997	55	58	483	92	82	69	66	20	15
Yeh (8)	Taiwan	1998	56	55	52	93	53	94	?	10	21
Lee (9)	Taiwan	2001	53	62	133	92	69	73	71		6
Alvarez (10)	Spain	2001	60	62	133	90	40	87	73		14
Tan (11)	Singapore	2005	60	58	80	?	?	76	?		4
Ferraioli (12)	Italy	2005	61	61	148	?	?	?	?		0
